# Optimization of Nitrogen, Phosphorus, and Potassium Fertilization Rates for Overseeded Perennial Ryegrass Turf on Dormant Bermudagrass in a Transitional Climate

**DOI:** 10.3389/fpls.2018.00487

**Published:** 2018-04-16

**Authors:** Muhammad Ihtisham, Shah Fahad, Tao Luo, Robert M. Larkin, Shaohua Yin, Longqing Chen

**Affiliations:** ^1^Key Laboratory of Horticultural Plant Biology, College of Horticulture and Forestry Science, Huazhong Agricultural University, Wuhan, China; ^2^College of Plant Science and Technology, Huazhong Agriculture University, Wuhan, China; ^3^Agricultural Department, The University of Swabi, Khyber Pakhtunkhwa, Pakistan; ^4^Southwest Engineering Technology and Research Center of Landscape Architecture (State Forestry Administration), Southwest Forestry University, Kunming, China

**Keywords:** turfgrass, overseeding, fertilizer optimization, central composite rotatable design, integrated turf performance, modeling

## Abstract

Bermudagrass [*Cynodon dactylon* (L.) Pers.] turf loss due to severe cold in transitional climates is a major concern. To overcome this problem, warm-season grass is often overseeded with a cool-season turfgrass. In this study, modeling and efficient nutrient management were used to evaluate this problem. A three-factor and five-level central composite rotatable design (CCRD) with a simulation of a regression model was used to optimize fertilization rates. The study investigated the combined effects of fertilization with nitrogen (N), phosphorus (P), and potassium (K) on both the morphological and physiological attributes and on the integrated turf performance (ITP) of overseeded perennial ryegrass (*Lolium perenne*). Fertilization with N and P significantly increased turf height, density, color, fresh and dry weights, while N, P, and K significantly affected turf cover, quality and winter-kill. The Spring transition was delayed by fertilization with N and P, and accelerated by fertilization with K. Photosynthesis (*Pn*), transpiration (*Tr*), and stomatal conductance (*Gs*) were considerably enhanced by fertilization with N, P, and K. Protein levels and total chlorophyll levels were substantially increased by fertilization with N and P and with N, P, and K, respectively, during a 2-year period. During two separate experiments conducted during 2 consecutive years, the optimal combinations of N, P, and K were N: 30, P: 24, K: 9, and N: 30, P: 27, K: 6 g m^−2^. The major conclusion of this study is that a balanced nutrient application utilizing N, P, and K is key to enhancing the winter performance of perennial ryegrass.

## Introduction

In transitional zone environments, it is difficult for both cool and warm-season grasses to perform well throughout the year (Jiang and Huang, 2000). In transitional zones, cool-season grasses are favored relative to warm-season grasses, because of the winter dormancy of warm-season grasses, which reduce turfgrass cover for an extended period of time. Bermudagrass is a warm-season turfgrass species used in sports fields, golf courses, parks, lawns, and prairies, because of its recuperative potential and extreme thermotolerance, however, low temperatures induce extended winter dormancy in bermudagrass (Fan et al., 2014). To overcome this problem, bermudagrass is commonly overseeded with a cool-season turfgrass (Shi et al., 2014a,b). The practice of winter overseeding usually involves seeding a cool-season grass into an established warm-season turfgrass for temporarily restoring turfgrass cover, color, quality, playability, aesthetics, functional quality, and overall performance during the dormant cool period (Pompeiano et al., 2011). Perennial ryegrass (*Lolium perenne* L.) is a major turf and forage grass, and commonly overseeded on bermudagrass (*Cynodon dactylon* L.) that is applied in the late summer through the mid-fall (Richardson, 2004; Verdenal et al., 2008; Yang et al., 2014). An ideal turfgrass for overseeding should quickly establish and maintain high turf quality. Additionally, an ideal turfgrass should not impede a smooth transition to bermudagrass in the spring (Beard and Menn, 1988).

An appropriate amount of nitrogen (N) fertilizer promotes photosynthesis, the production of dry matter, and stress tolerance (Guo et al., 2009). However, either under or over fertilization negatively affects plant growth (Hons et al., 2004). Deficiencies in phosphorus (P), another most important macronutrient negatively affects plant growth (Vance, 2001). P promotes winter survival, cold hardiness and dry matter production (Tyler et al., 1981). Similarly, potassium (K) fertilization plays a key role in turfgrass quality, root growth, disease resistance and cold hardiness (Snyder and Cisar, 2000).

Climatic changes (extreme low and high temperature) will cause instability and are anticipated to become more frequent. The low temperatures and short photoperiods of winter reduce grass production (Uleberg et al., 2014). These abiotic stresses adversely affect plant growth by inducing morphological, physiological, and biochemical changes in plants (Larcher, 2003). Inadequate fertilization enhances sensitivity to abiotic stress, even in highly managed turf surfaces (Pompeiano et al., 2011). Although the recommended nutrient rate for each crop is impractical for a number of reasons, and sessile nature of plants force agriculturists to develop more efficient fertilization programs for different seasons and localities.

Previous studies on turf grasses were limited to individual turfgrass species only in warm or cool seasons that tested the effects of nutrients in laboratories and nurseries. Unfortunately, studies on the quantitative relationship between fertilizers and turf quality are rarely reported, and limited research has been done on overseeding practices. There are few reports that investigated the combined effects of N, P, or K on cool-season turfgrasses, but to the best of our knowledge, no one has attempted to optimize the combination of N, P, and K on overseeded perennial ryegrass and quantify the interactive effects of these macronutrients on integrated turf performance (ITP).

Thus, the main objectives of this study were to quantify the interactive effects of N, P, and K fertilization on the morphology, physiology, and ITP of overseeded perennial ryegrass, and to develop a model that predicts the optimal combination of N, P, and K for maximum ITP.

## Methods

### Experimental site description and sample collection

**Figure 1:**
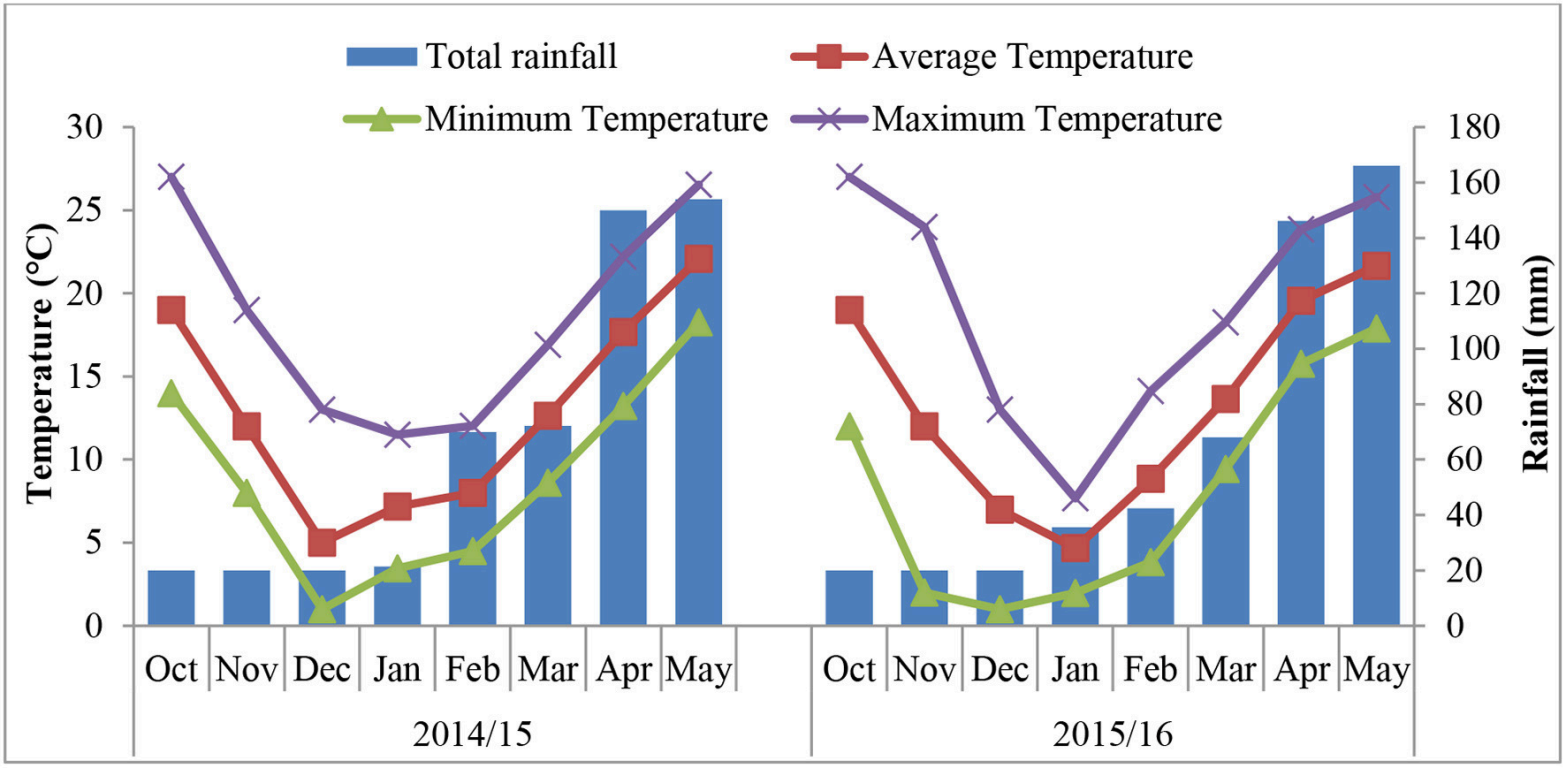
Monthly total rainfall and monthly maximum, minimum and mean temperature during the growing seasons of the two experiments.

The experiment was conducted from 2014 to 2016 at an ornamental plant garden at Huazhong Agricultural University (HZAU), Wuhan-China (30°28 N, 114°21 E). Meteorological data for both growing years were collected from a weather station located near the experimental site. This region has a humid subtropical monsoon climate, with a mean annual rainfall of 1,200–1,500 mm. From September of 2014 to May of 2015, the average annual temperature ranged from 5 to 22°C, while it was 4.6 to 21.6°C from September 2015 to May 2016 (Figure 1). There was snowfall in January and February during both years. Soil samples were taken randomly at a depth of 20 cm to test its physiochemical properties. The soil has a loess structure, pH 6.5, 19.80 g kg^−1^ organic matter content, total N of 1.39 g kg^−1^, available P of 25.56 mg kg^−1^, and available K of 151.14 mg kg^−1^.

### Field preparation and crop husbandry

Both experiments were initiated in May of 2014 and 2015. The field selected for experimentation was sprayed with glyphosate, a pre-emergence herbicide, and well plowed. Plant remnants, debris, and weeds were removed. A thick layer of washed sand was broadcasted and thoroughly mixed with a rotavator and leveled. A total of 24 plots with dimensions of 2 × 1.5 m (length × width) were arranged in 4 rows. Each row contained 6 plots. There were 50 cm distance between each row. This arrangement created 6 columns that contained 4 plots each. There were 60 cm distance between each column. Hybrid bermudagrass [*Cynodon dactylon* (L.) Pers.] “Tifway419” was plugged in the second week of May from one square inch plugs at a distance of 5–6 inches. Upon new growth, plots were fertilized with 10 g m^−2^ of N (urea, 46%), P (superphosphate, P_2_O_5_ ≥ 15%), and K (potassium sulfate, K_2_O ≥ 50%). Management practices like weeding, insect pest examination and irrigation were performed to maintain high quality turf for overseeding.

Overseeding experiments were conducted in the first week of October 2014 and 2015. Bermudagrass was vertically mowed in two directions to a height of <1 cm and slightly raked with brushes to ensure that seeds dropped in-to the soil. Perennial ryegrass (*Lolium perenne L.)* seeds were obtained from the ornamental plant nursery at HZAU and were overseeded at a density of 30 g m^−2^. Plots were irrigated with an automatic sprinkler, immediately after overseeding. After germination, the plots were fertilized with combinations of N, P, and K (Table 2).

Urea (N ≥ 46%), superphosphate (P_2_O_5_ ≥ 15%), and potassium sulfate (K_2_O ≥ 50%) were used as fertilizers. An additional 20 g N m^−2^ was also applied in late April of each year to promote the recovery of the bermudagrass.

### Data collection and measurements

#### Morphological data

Percent ryegrass cover was measured three times biweekly using the line-intersect method (Jellicorse et al., 2012) in the second week of November during both experiments. When the percent cover reached >80%, we analyzed turf height, density, color, and quality. Turf height was measured four times on five randomly selected tillers. Turf density based on average leaf counts was measured thrice on a monthly basis by uprooting five randomly selected plants. A biweekly visual rating scale (1–9) for turf color and quality was used 9 times. Winter-kill was measured quantitatively on a rating of 0–9. A 10 cm^2^ grid was randomly placed on the turf at three different dates in January of each year. The shoots within the grid were cut above the crowns and fresh weights were determined and then normalized to square decimeters (g/dm^2^). The same procedure was opted for aboveground dry weight except that samples were oven dried at 65°C for 48 h. For both experiments, the spring transition of bermudagrass from overseeded ryegrass was measured three times every week beginning in the first week of May using the line-intersect method.

#### Gas-exchange

Photosynthetic parameters, i.e., photosynthesis (*Pn*), transpiration (*Tr*), stomatal conductance (*gs*), inter cellular CO_2_ concentration (*Ci*), and water use efficiency (WUE) were measured by LI-6400 portable photosynthesis system (LI-COR Inc., Lincoln, NE, USA). Measurements were taken by holding multiple intact leaves of same culm in the chamber. The constant airflow rate was 500 μmol s^−1^, saturation PFD was 1,200 μmol m^−2^ s^−1^, the CO_2_ concentration was 350 cm^3^ m^−3^, and the temperature was 20°C with 80% relative humidity. Measurements were made during sunny days between 09:00 to 11:30 a.m. (Beijing time) on four different dates at one-week intervals during January 2015 and 2016. WUE is the ratio of *Pn* to *Tr*.

#### Protein and chlorophyll

Protein content in the fresh leaves of ryegrass was quantified using a spectrophotometer and a commercial assay kit (A045-2) from Nanjing Jiancheng Bioengineering Institute, China. Total chlorophyll content was quantified as previously described (Esmaeili et al., 2015).

#### Experimental design and statistical analysis

A three-factor (N, P, and K) and five-level central composite rotatable design (CCRD) was used for optimization and evaluation of the combined effects of N, P, and K on the morphology, physiology of the ryegrass, and the interactive effects on ITP. The actual amount of fertilizer varied between a minimum value of 0 and a maximum value of 30 g m^−2^. Treatment levels and ranges of independent variables in the experiment were coded (Table 1) according to the following Equation (1):

(1) $$\begin{array}{l} Xi=\frac{x_{i}-x_{i}^{0}}{\Delta x_{i}} \end{array}$$

where *Xi* is the dimensionless coded value of the *i*th variable, *xi* is the real value of the *i*th variable, *xi*^0^ is the real value of the *i*th variable at the center point, and Δ*xi* is the scaling factor (Montgomery, 2001). The independent variable limits and their values are shown in (Table 2).

**Table 1:** Independent variables, codes, and both their coded and actual values in the central composite rotatable design.

			**Coded and actual levels**				
**Independent variables**	**Codes**	**Symbols**	**−1.682**	**−1**	**0**	**1**	**1.682**
Nitrogen	X_1_	N	0	6.082	15	23.918	30
Phosphorus	X_2_	P	0	6.082	15	23.918	30
Potassium	X_3_	K	0	6.082	15	23.918	30

*X_1_, X_2_, and X_3_ represent codes for N, P, and K respectively. −1.682, −1, 0, 1, and 1.682 represent coded, and 0, 6.082, 15, 23.918, and 30 represent actual levels for N, P, and K respectively*.

**Table 2:** Experimental design and layout of treatment combinations in the central composite rotatable design.

**Treatments**	**Coded values**			**Actual amount of independent variables**		
	**X_1_**	**X_2_**	**X_3_**	**N (g/m^2^)**	**P (g/m^2^)**	**K (g/m^2^)**
1	1	1	1	23.918	23.918	23.918
2	1	1	−1	23.918	23.918	6.082
3	1	−1	1	23.918	6.082	23.918
4	1	−1	−1	23.918	6.082	6.082
5	−1	1	1	6.082	23.918	23.918
6	−1	1	−1	6.082	23.918	6.082
7	−1	−1	1	6.082	6.082	23.918
8	−1	−1	−1	6.082	6.082	6.082
9	1.682	0	0	30	15	15
10	−1.682	0	0	0	15	15
11	0	1.682	0	15	30	15
12	0	−1.682	0	15	0	15
13	0	0	1.682	15	15	30
14	0	0	−1.682	15	15	0
15	0	0	0	15	15	15
16	0	0	0	15	15	15
17	0	0	0	15	15	15
18	0	0	0	15	15	15
19	0	0	0	15	15	15
20	0	0	0	15	15	15
21	0	0	0	15	15	15
22	0	0	0	15	15	15
23	0	0	0	15	15	15
24				0	0	0

*Treatments 1 through 24 represent the total number of experimental plots. X_1_, X_2_, and X_3_ represent coded values for N, P, and K respectively*.

In the CCRD, the number of experimental units was based on a complete 2^k^ factorial. The total number of experimental runs was calculated with Equation (2)

(2) $$\begin{array}{l} T=2^{k}+2k+m_{0} \end{array}$$

where *T* is the number of experimental runs, *k* is the number of factors, and *m*_0_ is the number of replicates of the center point.

The center point of the CCRD needs to be replicated more than five times to accurately determine its value. In contrast, the other treatments do not need to be replicated (Montgomery, 2001). The center point treatment was replicated 9 times, with a control (runs = 2^3^ + 2 × 3 + 9 + 1 = 24).

Morphological, physiological, and ITP responses to N, P, and K fertilization ratios were fit to a second-order polynomial Equation (3)

(3) $$\begin{array}{l} Y=a_{0}+a_{1}x_{1}+a_{2}x_{2}+a_{3}x_{3}+a_{4}x_{1}x_{2}+a_{5}x_{1}x_{3}+a_{6}x_{2}x_{3}\\ +a_{7}x_{1}x_{1}+a_{8}x_{2}x_{2}+a_{9}x_{3}x_{3} \end{array}$$

where *Y* is the dependent variable; *a*_0_ is a constant; *a*_1_, *a*_2_, and *a*_3_ are linear coefficients of *x*_1_, *x*_2_, and *x*_3_, respectively; *a*_4_, *a*_5_, and *a*_6_ are interaction coefficients; *a*_7_, *a*_8_, and *a*_9_ are the quadratic coefficients of *x*_1_, *x*_2_, and *x*_3_, respectively; and *x*_1_, *x*_2_, and *x*_3_ are the coded values for N, P, and K respectively.

Linear regression of the above equation was performed for each variable. The constant, regression coefficient of linear, quadratic, and interaction terms were determined in SPSS (16.0). Significance of the linear, quadratic, and interaction forms was judged using an *F*-value at *P* < 0.05. The adequacy of the regression equations was evaluated using the coefficient of determination, *R*^2^. MS Excel 2016 and SPSS (16.0) were used for statistical analysis and modeling. Sigma plot (8.0) was used to generate 3D surface plots to show interactive effects on ITP.

## Results

### Percent ryegrass cover

Positive values for the parameter coefficients N (a_1_), P (a_2_), and K (a_3_) during the first experiment (2014–2015) and the second experiment (2015–2016) demonstrate the enhancing effect of these nutrients on ryegrass cover. The relative magnitude of absolute values was: a_1_ > a_2_ > a_3_ (Table 4). The lowest percent cover was observed in the control plots, 45.33 and 51.33% during both experiments (Table 3). In the first and second experiments, the highest percent cover was 84.33 and 80.33% (Table 3), which was observed with N, P, K = 23.918 and N, P, K = 15, respectively.

**Table 3:** Morphological performance of overseeded perennial ryegrass on bermudagrass in response to different treatment combinations of nitrogen, phosphorus, and potassium fertilizer in the experiments conducted from 2014 to 2015 and 2015 to 2016.

**Treatments**	**% Ryegrass cover**		**Turf height (cm)**		**Turf color**		**Turf quality**		**Turf density**		**Winter-kill**		**Fresh weight (g/dm**^**2**^**)**		**Dry weight (g/dm**^**2**^**)**		**Spring transition**	
	**2014–2015**	**2015–2016**	**2014–2015**	**2015–2016**	**2014–2015**	**2015–2016**	**2014–2015**	**2015–2016**	**2014–2015**	**2015–2016**	**2014–2015**	**2015–2016**	**2014–2015**	**2015–2016**	**2014–2015**	**2015–2016**	**2014–2015**	**2015–2016**
1	84.33	79.33	11.16	11.61	8.27	8.29	8.42	8.38	20.53	24.87	8.1	8	29.00	26.33	5.91	6.91	23.2	36.4
2	81.33	79.67	13.68	14.10	8.49	8.53	8.68	8.82	24.27	27.53	8.75	8.8	35.33	30.67	7.65	8.30	28.2	32.2
3	79.00	73.00	11.54	12.31	8.17	8.22	8.44	8.41	21.27	24.40	8.35	8.1	21.00	21.33	4.56	7.42	32.6	22.4
4	75.00	72.67	10.82	12.83	8.13	8.40	8.43	8.74	22.33	25.07	8.3	8.45	22.00	23.33	5.13	5.87	31.4	18.4
5	72.33	73.00	7.21	6.84	6.36	6.21	6.39	6.90	9.93	10.87	4.75	4.5	4.33	3.67	1.44	1.50	54.4	58
6	76.33	77.67	7.29	7.35	6.42	6.48	6.40	6.44	11.47	12.93	4.75	4.75	7.00	9.67	1.78	3.23	48.4	59.6
7	70.67	70.67	7.07	7.27	6.34	6.36	6.31	6.26	9.73	12.33	5	4.75	5.67	8.33	1.61	3.15	58.8	61.8
8	68.33	67.00	6.72	7.56	6.06	6.18	5.98	6.16	11.00	12.20	4.5	4.5	5.00	7.00	1.60	2.50	60.8	48.4
9	72.00	78.00	11.50	11.64	8.56	8.49	8.43	8.38	22.20	23.00	8.5	8.4	32.33	21.67	7.18	6.20	39.6	52.6
10	52.67	59.67	4.66	5.06	4.89	4.57	4.46	4.09	7.87	8.33	2.25	1.75	2.33	2.67	1.34	1.46	60.4	59.4
11	79.00	77.33	9.01	10.48	7.09	7.20	7.38	7.43	17.13	16.27	6.75	7	16.00	17.00	4.45	4.94	66.0	50.6
12	64.67	67.67	7.63	8.37	6.39	6.59	6.80	7.02	14.07	15.00	6	6.6	11.33	9.00	3.53	3.04	60.6	38.6
13	73.33	75.00	9.16	8.68	6.97	7.11	7.54	7.40	16.27	17.07	7.25	7	8.67	11.33	2.78	3.35	59.2	57.6
14	72.67	72.00	9.29	9.54	6.96	7.14	7.24	7.03	16.47	17.67	6.75	6	11.33	12.67	3.88	3.66	43.4	50
15	80.00	80.33	8.73	9.75	7.03	7.02	7.41	7.83	18.93	21.27	7.4	7.25	18.00	17.67	4.22	5.01	61.4	58.2
16	80.33	78.33	9.69	10.01	7.04	7.01	7.53	7.73	17.87	18.13	7.35	7.25	16.33	19.33	3.90	6.04	62.6	54.2
17	79.67	76.33	11.52	10.29	7.86	7.62	8.28	8.01	20.73	19.67	7.75	7.55	19.33	23.33	5.32	6.21	61.2	62.6
18	80.67	78.67	10.50	10.62	7.54	7.66	8.10	8.08	18.47	20.13	7.75	7.65	18.67	18.00	5.35	5.46	61.2	58.6
19	79.67	74.00	10.34	9.96	7.60	7.57	7.89	8.00	19.87	18.93	7	7.35	21.00	16.67	5.00	5.70	64.8	53.2
20	77.33	74.67	9.80	10.70	7.44	7.48	7.84	7.82	18.20	19.00	7.5	7.35	19.33	22.33	4.95	6.11	65.4	49.4
21	78.33	75.33	11.21	11.07	8.28	8.01	8.47	8.17	19.07	24.87	7.8	8.25	21.33	20.00	5.27	5.45	62.8	48.8
22	77.67	74.33	11.77	10.16	8.12	7.99	8.28	8.22	20.87	19.07	7.5	8.2	20.33	19.00	5.37	4.69	60.6	47.2
23	76.67	78.00	10.41	10.10	7.90	7.77	7.96	8.21	20.07	20.73	7.65	8	20.67	17.33	5.07	4.76	60.0	54.4
24	45.33	51.33	4.57	4.67	4.23	4.27	3.49	3.44	6.93	9.33	1.75	1.75	1.67	2.00	0.86	0.95	49.4	56.4

**Table 4:** Parameter coefficients of the regression equation (*Y* = *a*_0_ + *a*_1_*x*_1_ + *a*_2_*x*_2_ + *a*_3_*x*_2_+ *a*_4_*x*_1_*x*_2_ + *a*_5_*x*_1_*x*_3_ + *a*_6_*x*_2_*x*_3_ + *a*_7_$x_{1}^{2}$ + *a*_8_$x_{2}^{2}$ + *a*_9_$x_{3}^{2}$) for % ryegrass cover, turf height (cm), turf color, turf quality, turf density, winter-kill, fresh weight (g/dm^2^), dry weight (g/dm^2^), and spring transition during the first experiment (2014–2015) and the second experiment (2015–2016). *x*_1_, *x*_2_, *x*_3_ are the coded values of the fertilizer rates for N, P, and K.

**Y**	**Year**	**a_0_**	**a_1_**	**a_2_**	**a_3_**	**a_4_**	**a_5_**	**a_6_**	**a_7_**	**a_8_**	**a_9_**	***R*^2^**
% Ryegrass cover	2014–2015	75.30	4.72	3.32	0.47	0.25	1.08	−0.917	−4.32	−0.96	−0.548	0.78^**^
	2015–2016	74.46	3.45	3.12	0.30	0.04	0.12	−1.13	−2.18	−0.89	−0.53	0.77^**^
Turf height	2014–2015	9.59	2.23	0.40	−0.13	0.22	−0.26	−0.46	−0.63	−0.54	−0.22	0.87^**^
	2015–2016	9.84	2.41	0.25	−0.39	0.15	−0.28	−0.27	−0.46	−0.08	−0.19	0.90^**^
Turf color	2014–2015	7.30	1.03	0.15	0.00	0.01	−0.05	−0.08	−0.22	−0.21	−0.13	0.87^**^
	2015–2016	7.30	1.09	0.10	−0.04	0.01	−0.04	−0.06	−0.26	−0.13	−0.05	0.89^**^
Turf quality	2014–2015	7.51	1.14	0.12	0.04	−0.03	−0.07	−0.08	−0.44	−0.22	−0.11	0.92^**^
	2015–2016	7.55	1.16	0.12	0.03	−0.11	−0.17	0.03	−0.48	−0.13	−0.14	0.92^**^
Turf density	2014–2015	17.33	5.15	0.51	−0.58	0.07	−0.25	−0.37	−1.34	−1.14	−0.87	0.94^**^
	2015–2016	18.67	5.73	0.32	−0.46	0.46	−0.18	−0.52	−1.02	−1.04	−0.42	0.86^**^
Winter-kill	2014–2015	6.86	1.83	0.11	0.05	0.02	−0.14	−0.15	−0.68	−0.32	−0.10	0.97^**^
	2015–2016	6.85	1.91	0.07	0.04	0.03	−0.14	−0.12	−0.82	−0.21	−0.31	0.96^**^
Fresh weight	2014–2015	16.80	9.94	2.19	−1.01	2.58	−0.67	−1.08	−0.16	−1.45	−2.75	0.96^**^
	2015–2016	16.45	7.68	1.74	−0.97	1.79	−0.21	−1.21	−1.62	−1.33	−1.68	0.87^**^
Dry weight	2014–2015	4.23	1.95	0.40	−0.33	0.48	−0.25	−0.19	−0.26	−0.35	−0.59	0.95^**^
	2015–2016	4.82	1.91	0.31	−0.11	0.36	0.15	−0.67	−0.34	−0.28	−0.46	0.85^**^
Spring transition	2014–2015	53.34	−10.4	−1.49	1.96	0.52	−0.98	0.23	−6.85	−2.15	−6.39	0.77^*^
	2015–2016	49.24	−9.51	4.05	2.40	2.55	−0.45	−1.85	−1.22	−5.25	−1.99	0.67^*^

** *Significance at P < 0.01*.

* *Significance at P < 0.05. NS, Not Significant*.

### Turf height

For turf height, the coefficients a_1_ and a_2_ were positive with a_1_ > a_2_. In contrast, the coefficient a_3_ was negative during both years (Table 4). The lowest turf heights were recorded in the control plots (Table 3) during both experiments and were 4.57 and 4.67 cm. In both experiments, the highest turf heights of 13.68 and 14.10 cm (Table 3) were observed when N, P = 23.918 and K = 6.082.

### Turf density

N and P enhanced the turf density 10-fold in the first experiment and 17-fold in the second experiment and a_1_ > a_2_. In contrast, K had no effect (Table 4). The highest turf density of 24.27 and 27.53 were achieved at N, P = 23.918, K = 6.082 treated plots during both experiments (Table 3). In the first experiment, the control treatment and in the second experiment, the N = 0 and P, K = 15 treatment yielded the lowest turf densities of 6.93 and 8.33, respectively (Table 3).

### Turf color

The parameter coefficients for turf color a_1_ (maximum among all) and a_2_ were positive during the study period. In contrast, a_3_ (minimum among all) was positive and negative in consecutive years. (Table 4). Poorest color ratings of 4.23 and 4.27 were observed in the control plots (Table 3). We observed the highest color coefficients of 8.56 and 8.53 (Table 3) in response to the N = 30 and P, K = 15 treatment during the first experiment and in response to the N, P = 23.918 and K = 6.082 treatment during the second experiment.

### Turf quality

Turf quality of the overseeded ryegrass was similar during both experiments. In other words, a_1_, a_2_, and a_3_ were all positive with N having the greatest effect, followed by P and then K (Table 4). The N, P = 23.918 and K = 6.028 treatment yielded the highest turf quality of 8.68 and 8.82, and the control plots yielded the lowest turf quality of 3.49 and 3.44 during both years (Table 3).

### Winter-kill

Throughout both experiments, the ryegrass survived well during winter before the onset of snow fall. The worst winter-killings were detected in unfertilized or zero N plots (Table 3). In general, all of the three factors ameliorated the worst effects of low temperature on ryegrass (Table 4), with the relative magnitudes of the absolute values rank as follows: a_1_ > a_2_ > a_3_.

### Fresh and dry weight (g dm^−2^)

During both experiments, the a_1_ and a_2_ coefficients were positive and a_1_ > a_2_. In contrast, the a_3_ coefficient was negative (Table 4). For both the fresh and dry weights, the highest yields from both experiments were obtained from the N, P = 23.918 and K = 6.082 treated plots (Table 3). The lowest yields were obtained from the control plots during both experiments (Table 3).

### Spring transition

For the spring transition, in the first experiment, a_1_ and a_2_ were negative, and a_3_ was positive (Table 4). For the second experiment, a_1_ was negative and both a_2_ and a_3_ were positive. The spring transition to bermudagrass was generally more effective when fertilization was reduced.

### Gas-exchange, protein, and total chlorophyll

We analyzed a variety of physiological parameters for the ryegrass under different fertilization treatments (Table 5) and calculated regression coefficients for each parameter (Table 6). All coefficients for *Pn* were positive. The highest values we obtained for *Pn* were 6.25 and 4.27 (mmol m^−2^ s^−1^) from the N, P, K = 23.918 and N, P = 23.918, K = 6.082 treatments, respectively. The lowest values obtained for *Pn* were 0.76 and 0.72 (mmol m^−2^ s^−1^) from the control conditions during both years. Absolute values showed that N was more effective than P and K. The regression equation coefficients for *Tr* were positive. We observed a maximum of 2.32 and 1.29 (mmol m^−2^ s^−1^) from the N, K = 23.918, P = 6.082, and N = 23.918, P, K = 6.082 treatments, respectively, and a minimum of 0.16 and 0.17 (mmol m^−2^ s^−1^) from the control conditions. We observed a similar pattern for *Ci* and *Gs*. The maximum values for *Ci* 319.36 and 292.05 (mmol mol^−1^) and *Gs* 0.37 and 0.13 (mol m^−2^ s^−1^) in the N, K = 23.918, P = 6.082 conditions, and the lowest values for *Ci* and *Gs* in the control conditions (Table 5). Although a_1_ (N) and a_3_ (K) positively influenced *Ci* in the first experiment, only a_1_ (N) effectively improved *Ci* in the second experiment. *Gs* was positively affected by all the three factors in the first experiment, whereas only a_2_ (P) negatively affected in the second experiment. In contrast to these data, we observed the highest values for WUE in the treatments with lower quantities of fertilizer and lowest values for WUE in the treatments with higher quantities of fertilizer.

**Table 5:** Physiological analysis of perennial ryegrass that was overseeded on bermudagrass using different treatment combinations of nitrogen, phosphorus, and potassium fertilizers in two experiments (i.e., 2014–2015 and 2015–2016).

**Treatments**	***Pn*** **(mmol m**^**−2**^ **s**^**−1**^**)**		***Tr*** **(mmol m**^**−2**^ **s**^**−1**^**)**		***Ci*** **(mmol mol**^**−1**^**)**		***Gs*** **(mol m**^**−2**^ **s**^**−1**^**)**		**WUE (*****pn/Tr*****)**		**Protein (g L**^**−1**^**)**		**Total chlorophyll (mg Chl g**^**−1**^ **fw)**	
	**2014–2015**	**2015–2016**	**2014–2015**	**2015–2016**	**2014–2015**	**2015–2016**	**2014–2015**	**2015–2016**	**2014–2015**	**2015–2016**	**2014–2015**	**2015–2016**	**2014–2015**	**2015–2016**
1	6.25	3.64	1.44	0.95	288.84	277.83	0.25	0.09	4.35	3.84	2.09	2.11	1.60	1.72
2	4.95	4.27	2.11	1.00	309.69	279.15	0.33	0.10	2.34	4.26	2.87	2.40	1.55	1.91
3	3.70	3.42	2.32	1.18	319.36	292.05	0.37	0.13	1.59	2.91	1.75	1.90	1.57	1.70
4	4.17	3.83	1.54	1.29	298.30	277.70	0.28	0.10	2.70	2.97	2.58	1.93	1.64	1.82
5	2.54	2.09	0.69	0.61	281.15	225.96	0.06	0.03	3.69	3.41	0.91	0.68	1.06	1.16
6	3.58	2.98	0.70	0.43	266.13	251.31	0.10	0.04	5.11	6.90	1.46	1.20	1.00	1.09
7	1.65	2.10	0.36	0.54	308.52	266.63	0.07	0.04	4.65	3.86	0.99	0.86	0.96	1.05
8	1.96	2.02	0.35	0.27	287.42	251.27	0.06	0.06	5.63	7.54	1.51	0.94	0.89	0.96
9	4.53	3.14	1.41	1.23	258.40	274.28	0.19	0.12	3.22	2.56	2.42	1.56	1.90	1.80
10	1.29	1.12	0.17	0.18	231.77	198.29	0.04	0.02	7.57	6.32	1.17	0.76	0.92	0.95
11	3.16	3.48	0.45	0.88	273.56	291.05	0.08	0.09	7.06	3.96	1.30	1.17	1.57	1.61
12	2.53	2.70	0.28	0.70	251.67	276.52	0.09	0.11	8.92	3.84	1.07	0.94	1.40	1.49
13	3.47	3.33	0.44	0.96	249.30	263.88	0.08	0.09	7.94	3.45	2.27	1.95	1.37	1.42
14	1.97	2.19	0.23	0.83	269.79	271.59	0.05	0.06	8.55	2.65	1.38	1.12	1.36	1.40
15	3.52	2.89	0.55	0.72	274.11	266.46	0.17	0.11	6.43	4.01	1.02	1.28	1.43	1.55
16	3.51	3.79	0.57	0.99	290.53	281.66	0.12	0.10	6.11	3.83	0.96	1.51	1.48	1.45
17	3.92	3.64	0.83	0.88	300.02	288.35	0.20	0.12	4.72	4.13	1.23	1.17	1.55	1.54
18	3.97	3.36	0.82	1.05	313.56	287.26	0.21	0.12	4.87	3.21	0.81	1.41	1.49	1.48
19	3.07	3.65	0.68	0.92	315.39	287.17	0.13	0.11	4.53	3.95	1.30	1.15	1.44	1.53
20	3.48	4.07	0.67	0.93	297.94	247.62	0.14	0.09	5.21	4.39	0.94	1.69	1.56	1.60
21	4.18	3.90	1.00	1.04	292.65	245.54	0.19	0.12	4.20	3.75	1.38	1.54	1.60	1.62
22	4.05	3.59	0.79	0.96	269.44	268.88	0.13	0.12	5.11	3.73	1.36	1.41	1.56	1.47
23	3.98	3.50	0.94	1.07	267.99	266.05	0.21	0.11	4.21	3.28	1.25	1.56	1.59	1.58
24	0.76	0.72	0.16	0.17	209.97	194.97	0.03	0.02	4.66	4.25	0.26	0.18	0.82	0.89

**Table 6:** Parameter coefficients of the regression equation (*Y* = *a*_0_ + *a*_1_*x*_1_ + *a*_2_*x*_2_ + *a*_3_*x*_2_+ *a*_4_*x*_1_*x*_2_ + *a*_5_*x*_1_*x*_3_ + *a*_6_*x*_2_*x*_3_ + *a*_7_$x_{1}^{2}$ + *a*_8_$x_{2}^{2}$ + *a*_9_$x_{3}^{2}$) for photosynthesis (*Pn*), transpiration (*Tr*), inter cellular CO_2_ concentration (*Ci*), stomal conductance (*Gs*), water use efficiency (WUE), protein levels and total chlorophyll during the first experiment (2014–2015) and the second experiment (2015–2016). *x*_1_, *x*_2_, *x*_3_ are the coded values of the fertilizer rates for N, P, and K.

**Y**	**Year**	**a_0_**	**a_1_**	**a_2_**	**a_3_**	**a_4_**	**a_5_**	**a_6_**	**a_7_**	**a_8_**	**a_9_**	***R*^2^**
*Pn*	2014–2015	3.45	1.08	0.50	0.14	0.10	0.27	0.12	−0.12	−0.14	−0.19	0.78^**^
	2015–2016	3.16	0.68	0.21	0.00	−0.04	−0.03	−0.15	−0.43	−0.09	−0.21	0.89^*^
*Tr*	2014–2015	0.84	0.54	0.05	0.03	−0.13	0.01	−0.18	0.16	0.01	0.00	0.67^*^
	2015–2016	0.85	0.32	0.00	0.04	−0.09	−0.08	0.00	−0.09	−0.06	−0.02	0.96^**^
*Ci*	2014–2015	283.28	8.62	−2.27	0.14	3.69	−4.49	−6.00	−7.76	−1.57	−2.65	0.23NS
	2015–2016	266.80	18.99	−2.12	−0.73	3.48	2.88	−7.05	−11.62	5.17	−0.50	0.74^*^
*Gs*	2014–2015	0.15	0.09	0.00	0.00	−0.01	0.01	−0.03	0.01	−0.01	−0.01	0.63NS
	2015–2016	0.09	0.03	−0.01	0.00	0.00	0.01	−0.01	−0.02	−0.01	−0.02	0.89^**^
WUE	2014–2015	5.16	−1.13	−0.16	−0.18	0.48	0.41	0.34	−0.62	0.30	0.39	0.39*NS*
	2015–2016	4.03	−1.03	0.10	−0.46	0.41	0.84	−0.02	0.36	0.17	−0.13	0.88^**^
Protein	2014–2015	1.48	0.48	0.07	−0.09	0.09	−0.07	0.00	0.26	0.05	0.27	0.77^**^
	2015–2016	1.40	0.44	0.08	0.04	0.08	0.04	−0.09	−0.04	−0.07	0.10	0.70^*^
Total chlorophyll	2014–2015	1.41	0.30	0.03	0.01	−0.03	−0.02	0.01	−0.07	−0.04	−0.08	0.94^**^
	2015–2016	1.47	0.32	0.04	−0.01	−0.02	−0.06	−0.01	−0.06	0.00	−0.05	0.95^**^

** *Significance at P < 0.01*.

* *Significance at P < 0.05. NS, Not Significant*.

We observed highest concentration of protein 2.87 and 2.40 (g L^−1^) in extracts prepared from the N, P = 23.918, K = 6.082 treated plants and that of lowest concentrations of protein 0.26 and 0.18 (g L^−1^) in the control conditions. Regression coefficients indicated that although N and P consistently boosted protein concentration, K fertilization had no significant effects in the first experiment.

Total chlorophyll content in leaves was effectively enhanced by the NPK fertilization (first experiment) and NP fertilization (second experiment). N fertilization enhanced chlorophyll content the most (Table 6). We observed the maximum levels of chlorophyll 1.90 and 1.91 (mg Chl g^−1^ fw) in response to the N = 30, P, K = 15 and N, P = 23.918, K = 6.082 treatments and the lowest levels of chlorophyll 0.82 and 0.18 (mg Chl g^−1^ fw) in the control conditions (Table 5).

### Effects of N, P, and K on integrated turf performance (ITP)

The data in (Table 3) was transformed according to the experimental design using Equation (4) yielding (Tables 7, 8).

(4) $$\begin{array}{l} X=\frac{x-x_{min}}{{x_{max}-\;x}_{min}} \end{array}$$

**Table 7:** Transformed values of morphological parameters according to the central composite rotatable design and integrated turf performance (ITP) of overseeded perennial ryegrass on bermudagrass from 2014 to 2015.

**Treatments**	**% Ryegrass cover**	**Turf height (cm)**	**Turf color**	**Turf quality**	**Turf density**	**Winter kill**	**Fresh weight (g/dm^2^)**	**Dry weight (g/dm^2^)**	**Spring transition**	**Integrated turf performance (Y)**
Max/Min	95/35	18/1	9/0	9/0	30/3	9/0	40/1	10/0.2	80/10	
1	0.822	0.597	0.918	0.935	0.649	0.9	0.717	0.582	0.188	0.701
2	0.772	0.745	0.943	0.964	0.787	0.972	0.88	0.76	0.26	0.787
3	0.733	0.62	0.907	0.938	0.676	0.927	0.512	0.444	0.322	0.675
4	0.666	0.577	0.903	0.937	0.716	0.922	0.538	0.503	0.305	0.674
5	0.622	0.365	0.706	0.709	0.256	0.527	0.085	0.126	0.634	0.448
6	0.688	0.369	0.713	0.711	0.313	0.527	0.153	0.161	0.548	0.465
7	0.594	0.357	0.704	0.701	0.249	0.555	0.119	0.144	0.697	0.458
8	0.555	0.336	0.67	0.664	0.296	0.5	0.102	0.142	0.725	0.444
9	0.616	0.617	0.95	0.937	0.711	0.944	0.803	0.712	0.422	0.746
10	0.294	0.215	0.543	0.495	0.18	0.25	0.034	0.116	0.72	0.316
11	0.733	0.471	0.787	0.819	0.523	0.75	0.384	0.434	0.8	0.633
12	0.494	0.39	0.709	0.755	0.409	0.667	0.264	0.34	0.722	0.528
13	0.638	0.479	0.774	0.838	0.491	0.805	0.196	0.263	0.702	0.576
14	0.627	0.487	0.772	0.804	0.498	0.75	0.264	0.375	0.477	0.562
15	0.75	0.454	0.781	0.823	0.59	0.822	0.435	0.41	0.734	0.644
16	0.755	0.51	0.782	0.837	0.55	0.816	0.393	0.377	0.751	0.641
17	0.744	0.618	0.872	0.919	0.656	0.861	0.471	0.522	0.731	0.71
18	0.761	0.558	0.838	0.9	0.572	0.861	0.452	0.525	0.731	0.689
19	0.744	0.549	0.844	0.876	0.624	0.777	0.512	0.489	0.782	0.689
20	0.705	0.517	0.827	0.871	0.562	0.833	0.471	0.484	0.791	0.673
21	0.722	0.601	0.919	0.94	0.595	0.866	0.521	0.517	0.754	0.715
22	0.711	0.633	0.902	0.919	0.661	0.833	0.495	0.527	0.722	0.711
23	0.694	0.553	0.877	0.883	0.632	0.85	0.504	0.497	0.714	0.689

**Table 8:** Transformed values of morphological parameters according to the central composite rotatable design and integrated turf performance (ITP) of overseeded perennial ryegrass on bermudagrass from 2015 to 2016.

**Treatments**	**% Rye grass Cover**	**Turf height (cm)**	**Turf color**	**Turf quality**	**Turf density**	**Winter kill**	**Fresh weight (g/dm^2^)**	**Dry weight (g/dm^2^)**	**Spring transition**	**Integrated turf performance (Y)**
Max/Min	95/35	18/1	9/0	9/0	30/3	9/0	40/1	10/0.2	80/10	
1	0.738	0.624	0.921	0.931	0.809	0.888	0.649	0.684	0.377	0.736
2	0.744	0.77	0.948	0.98	0.908	0.977	0.76	0.826	0.317	0.803
3	0.633	0.665	0.913	0.934	0.792	0.9	0.521	0.736	0.177	0.697
4	0.627	0.695	0.933	0.971	0.817	0.938	0.572	0.578	0.12	0.695
5	0.633	0.343	0.69	0.766	0.291	0.5	0.068	0.132	0.685	0.456
6	0.711	0.373	0.719	0.716	0.367	0.527	0.222	0.309	0.708	0.517
7	0.594	0.368	0.706	0.695	0.345	0.527	0.188	0.301	0.74	0.496
8	0.533	0.385	0.686	0.683	0.341	0.5	0.153	0.234	0.548	0.451
9	0.716	0.625	0.943	0.931	0.741	0.933	0.529	0.612	0.608	0.737
10	0.411	0.238	0.507	0.454	0.197	0.194	0.042	0.128	0.705	0.32
11	0.705	0.557	0.8	0.825	0.491	0.777	0.41	0.484	0.58	0.625
12	0.544	0.433	0.732	0.78	0.444	0.733	0.205	0.29	0.408	0.507
13	0.666	0.451	0.79	0.822	0.521	0.777	0.264	0.321	0.68	0.588
14	0.616	0.502	0.793	0.781	0.543	0.666	0.299	0.353	0.571	0.569
15	0.755	0.514	0.78	0.87	0.676	0.805	0.427	0.491	0.688	0.667
16	0.722	0.529	0.779	0.859	0.56	0.805	0.47	0.595	0.631	0.661
17	0.688	0.546	0.846	0.89	0.617	0.839	0.572	0.613	0.751	0.707
18	0.727	0.565	0.85	0.897	0.634	0.85	0.435	0.536	0.694	0.688
19	0.65	0.526	0.84	0.889	0.59	0.816	0.401	0.561	0.617	0.654
20	0.661	0.57	0.831	0.869	0.592	0.816	0.547	0.602	0.562	0.672
21	0.672	0.592	0.89	0.907	0.809	0.916	0.487	0.535	0.554	0.707
22	0.655	0.538	0.887	0.913	0.595	0.911	0.461	0.457	0.531	0.661
23	0.716	0.535	0.862	0.912	0.656	0.889	0.418	0.464	0.634	0.676

In Equation (4), *X* is the coded value for each treatment, *x* is the measured value of each treatment, *x*_*min*_ is the minimum, and x_max_ is the maximum value recorded for each parameter.

The ITP of perennial ryegrass was best (0.787 and 0.803) when treated with medium-high N, P and medium-low K (N, P = 23.918 and K = 6.082). The ITP was the worst (0.316 and 0.320) when the perennial ryegrass was treated with zero N and medium P and K (N = 0, P, K = 15) during both experiments. These data indicate that N-containing fertilizer has a substantial effect on overall turf quality (Tables 7, 8).

### Regression model of ITP under N, P, and K

We applied regression analysis to the values we obtained for ITP from the first experiment and a quadratic regression model Equation (5) was generated in which Y represents ITP.

(5) $$\begin{array}{l} Y=0.617+0.128x_{1}+0.024x_{2}-0.005x_{3}+0.016x_{1}x_{2}\\ -0.01x_{1}x_{3}-0.015x_{2}x_{3}-0.048x_{1}^{2}-0.031x_{2}^{2}-0.035x_{3}^{2} \end{array}$$

In Equation (5), the terms x_1_, x_2_, ${\text{x}}_{1}^{2}$, ${\text{x}}_{2}^{2}$, and ${\text{x}}_{3}^{2}$ were highly significant.

Using the same protocol, we generated a regression model Equation (6) for second experiment:

(6) $$\begin{array}{l} Y=0.622+0.125x_{1}+0.027x_{2}-0.004x_{3}+0.015x_{1}x_{2}\\ -0.06x_{1}x_{3}-0.022x_{2}x_{3}-0.041x_{1}^{2}-0.028x_{2}^{2}-0.023x_{3}^{2} \end{array}$$

In Equation (6), the terms x_1_, x_2_, x_2_x_3_, ${\text{x}}_{1}^{2}$, ${\text{x}}_{2}^{2}$, and ${\text{x}}_{3}^{2}$ were highly significant.

The coefficient of determination r^2^ (0.98 and 0.97) of the regression Equations (5) and (6), respectively, were significant with *p* ≤ 0.001. This shows that the *r*^2^ of the regression equations were highly significant during both years, signifying that the models accurately predicted ITP and that approximately 98 and 97% of the variations in the responses could be explained by the models. The lower value of “*P*” (probability) indicates a highly significant regression model (Halder et al., 2015).

### Interactive effects on ITP

**Figure 2:**
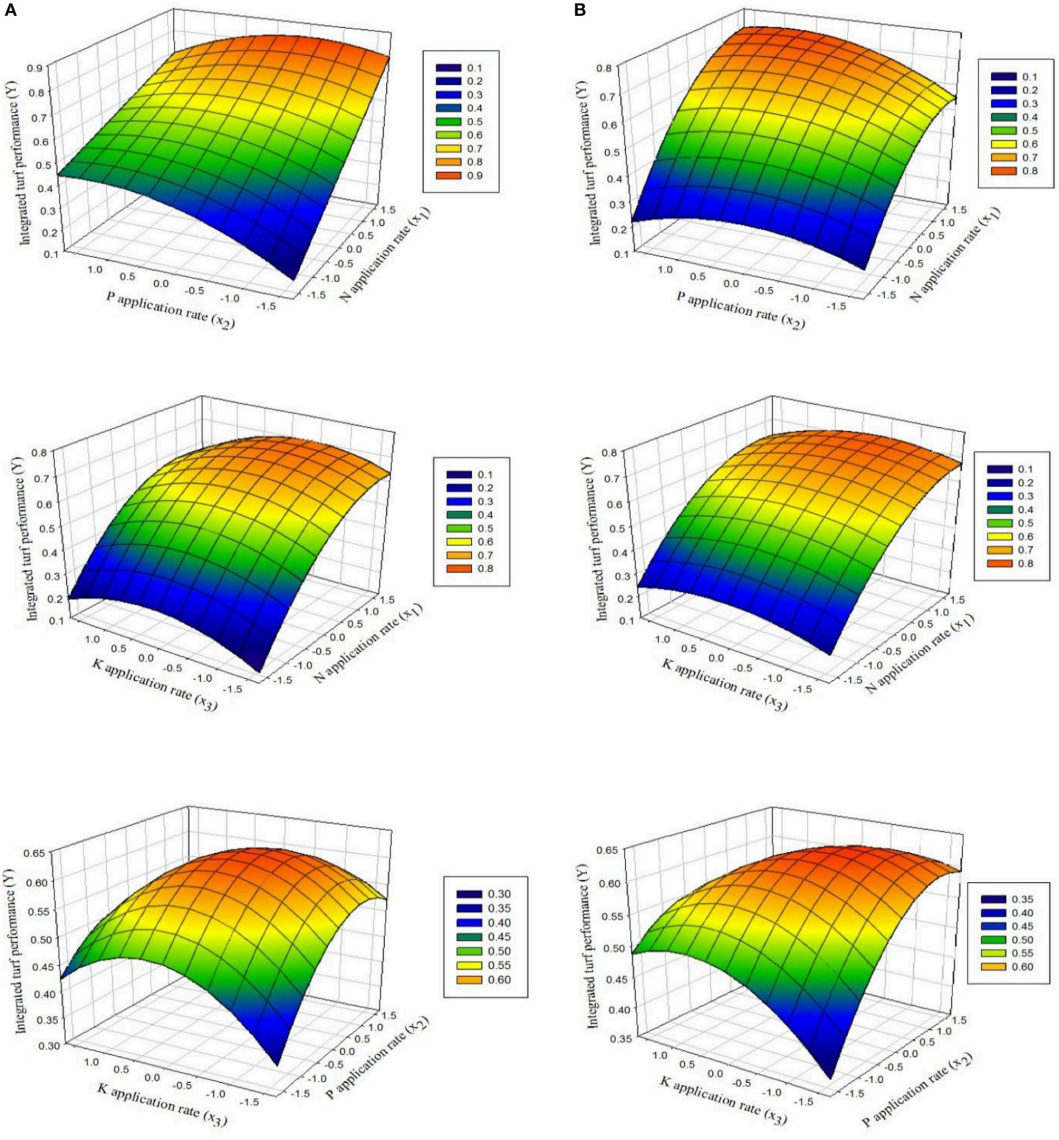
Response surfaces showing the effects of fertilization with N and P, N and K, and P and K on ITP for overseeded perennial ryegrass in **(A)** the first experiment and **(B)** the second experiment.

To investigate the interactive effects of the three factors using CCRD, 3D surface plots were generated by setting one variable at zero in the regression models (Equations 5 and 6). The interaction between the two factors (x and y) with ITP at the z-axis is shown in (Figure 2) for both experiments.

### Effect of x_1_ (N) and x_2_ (P)

Figure 2 shows that ITP first increased and then decreased as N and P increased. The optimal position for ITP is where its value remained constant. The effect of N fertilizer on ITP was obvious for both experiments. ITP rapidly increased as N fertilizer increased either at lower or high P rates followed by decline with further increase in N fertilizer. On the other hand, at lower N rates the increasing rates of P did not markedly increase ITP, whereas at higher rates of N with increasing P rates, the ITP increased distinctly, followed by gradual decline with consistent increase in N rates.

### Effect of x_1_ (N) and x_3_ (K)

The response surface plots of N and K fertilization in Figure 2 indicate that at lower amount of K, incremental increase in N led to rapid increases in ITP. When the amount of K was high, ITP first increased and then decreased with a continuous increase in N. In contrast, at low levels of N and increased levels of K, the lowest ITP was recorded. ITP reached a maximum at high levels of N and increased levels of K. Further increases in the levels of N and K induced decreases as indicated by red-colored and shaded areas (Figure 2).

### Effect of x_2_ (P) and x_3_ (K)

The interactive effects of fertilization with P and K depended on the amounts of each nutrient. ITP slightly increased at low levels of P and increasing levels of K, whereas it gradually decreases for K added above a particular level. In contrast, when P was added at high levels, the maximum values for ITP were obtained with increasing amounts of K up to a particular range. On the other hand, when low levels of K were added and the amounts of P were also increasing, ITP markedly increased. However, when K was added at high levels, the ITP decreased when P was increased consistently.

According to the qualitative analysis of factor interactions, it is clear that N is the most important nutrient, followed by P and K, respectively. To achieve a higher ITP, N fertilizer should be applied in high amounts, P fertilizer in medium to high amounts, and K fertilizer in low amounts.

### Optimization

The optimization of N, P, and K fertilization was performed using regression models (Equations 5 and 6) based on a computer simulation of the formula according to the regression model, within the range of each variable [−1. 682~1. 682], based upon a step length of “0.3346.” A total of 1,331 combinations and their corresponding ITP values were obtained, with the maximum value of 0.72 and 0.75 in first and second experiment, respectively, which corresponded to actual fertilizer values of N: 30, P: 24, K: 9, and N: 30, P: 27, K: 6 g m^−2^, respectively. The lowest ITP values of 0.02 and 0.07 were obtained in the control conditions during both experiments.

## Discussion

Nutrients, especially N, P, and K play a vital role in plant growth and production, as they are involved in many biological processes (Deng et al., 2014). The vegetative growth of perennial ryegrass is vigorous and hence, perennial ryegrass has high nutritional demands (Turgeon, 2008). The current study confirms that there are substantial effects of N, P, and K fertilization on the morphology, physiology, and ITP of overseeded perennial ryegrass on bermudagrass during the winter. During this study, the effect of N was more obvious followed by P and then K, (i.e., N>P>K).

In this study, we observed that applying N, P, and K fertilizer increased the percent of ryegrass cover in two distinct experiments (Table 4). The fact that N, P, and K are major macronutrients nutrients that are required for growth and development may explain these findings. N and P are required for the biosynthesis of abundant and essential biomolecules, such as proteins and nucleic acids, respectively, while K is the major cation of plant cells. Indeed, N-deficient soils often limit plant growth (Rajasekar et al., 2017). Consistent with our findings, fertilization with N, P, and K was previously reported to increase grass cover (Rodriguez et al., 2002; Guertal and Evans, 2006). We observed that fertilization with N and P significantly increased turf height and density, which defines the growth speed and the winter survival rate. A higher growth rate from tillers is a highly desirable turfgrass trait that improves aesthetics, functionality, and recovery from stress (Turgeon, 2008). We demonstrated that fertilization with N and P promoted higher rates of photosynthesis, which drives higher growth rates. Earlier reports demonstrated that the interactive effects of N and P enhanced that uptake and efficiencies of N and P in rice, wheat and grasses (Aulakh and Malhi, 2005) and documented similar findings in turfgrasses (Ahmad et al., 2003; Schlossberg and Schmidt, 2007).

Fertilization with N was major factor promoting the accumulation of chlorophyll in both experiments. In contrast, fertilization with P and K only promoted the accumulation of chlorophyll in the first experiment. Fertilization with N probably promotes the accumulation of chlorophyll in part because N is a major component of chlorophyll (Munshaw et al., 2007; Çelebi et al., 2010). Indeed, N-deficient plants typically exhibit chlorophyll-deficient phenotypes. K and P are not components of chlorophyll. In the first experiment, K and P probably promoted increases in the levels of chlorophyll by an indirect mechanism that is related to the general growth promoting properties of these important macronutrients. Our finding that fertilization with P and K has a minor effect on the accumulation of chlorophyll in turfgrass is consistent with previous work (Baldi et al., 2013). Consistent with previous work (Snyder and Cisar, 2000; McCarty and Miller, 2002), we found that fertilization with N, P, and K affected turf quality, which is an index that considers density, color, growth, and stress tolerance. Fertilization with N, P, and K protected ryegrass from cold stress (i.e., winter-kill). Indeed, plots that received higher rates of these macronutrients performed well. Our finding that fertilization with N, P, and K induced increases in growth during periods of cold stress might be explained by increases in water potential. The maintenance of water potential under high N conditions that included P and K was previously reported to promote leaf processes during stress conditions (Abid et al., 2016). Additionally, high rates of fertilization with N, P, and K were previously reported to promote winter hardiness (Razmjoo and Kaneko, 1993).

Fresh and dry weight of overseeded ryegrass increased as the amounts of N and P fertilizer increased. The increased shoot biomass was mainly due to enhanced lateral growth (i.e., density and height), as discussed above, that maximized leaf area thereby promoting the conversion of solar energy into chemical energy, which is essential for the production of biomass. Photosynthesis is the key driving force for the accumulation of dry matter, organ formation, and plant production (Zlatev and Lidon, 2012). The increase in the leaf area index, leaf photosynthetic area, and the accumulation of carbohydrates enhanced overall plant biomass (Din et al., 2017). Indeed, fertilization with N and P was reported to increases the accumulation of fresh and dry matter (Snyder and Cisar, 2000; Rodriguez et al., 2002; Stanford et al., 2005; Guertal, 2006).

High levels of fertilization attenuated the transition from ryegrass in the winter to bermudagrass in the spring. Generally, smooth, and rapid transitions occurred in plots that received lower applications of N and higher applications of both P and K. One explanation might be that because of the broad impact of N on plant growth and development, high N enhances the vigor of ryegrass by promoting processes that drives growth, such as photosynthesis, and promotes various types of stress tolerance, such as thermotolerance. Indeed, difficulties in the spring transition were previously attributed to the enhanced thermotolerance of new perennial ryegrass cultivars (Mazur and Wagner, 1987). The reduced availably of P and K, vertical mowing, the increase in temperature, and the additional N application in late April enhanced the recovery of bermudagrass. Consistent with these data, K fertilization was previously reported to promote an early spring transition (Duble, 2001; Webster and Ebdon, 2005).

Cold stress often decreases photosynthesis because of a variety of factors, such as stomatal behavior, photoinhibition, and the inhibition of carbon fixation (Wise et al., 2004). Fertilizers containing N, P, and K are well known for affecting photosynthesis in leaves (Wang et al., 2008). Deficiency and excess quantities of these fertilizers decrease the capacity of photosynthesis (Ashraf et al., 2001; Cechin and de Fátima Fumis, 2004). In general, fertilization with N, P, and K induced increase in the gas-exchange parameters of overseeded ryegrass during the winter. Higher rates of transpiration and the regulation of stomatal opening may induce the rate of photosynthesis. Greater biomass, elevated chlorophyll and higher protein content that were observed in response to NPK fertilization might be other factors that drove increases in *Pn, Tr*, and *Gs* (Alderman et al., 2011; Grygierzec, 2012). P is a vital structural part of nucleic acids (Wang et al., 2013) and promotes photosynthesis (Fredeen et al., 1990). Elevated levels of P could promote photosynthesis by contributing to the production of the products of noncyclic electron transport, adenosine triphosphate (ATP) and NADPH (Giersch and Robinson, 1987). K was suggested to induce increases in photosynthesis by regulating transpiration, stomatal behavior and turgor pressure (Römheld and Kirkby, 2010).

Elevated values of *Ci* were due to increased *Pn* and *Gs*. These data indicate that overseeded ryegrass leaves were capable of enhancing CO_2_ influx and fixing CO_2_ into carbohydrates. K fertilization regulates stomatal functions and N fertilization increases *Pn*. We suggest that acting together, these nutrients induce increases in CO_2_ fixation. Higher levels of N, P, and K reduced the WUE. The coefficient values indicated that P increased the WUE only in the second experiment. In our study, we found that increases in *Tr* were functions of N and K and that increases in *Tr* reduced the WUE. The lower WUE in the high-nutrient treated plot could also be attributed to lower increases in *Pn* relative to *Tr* (Fahad et al., 2016).

Protein and total chlorophyll were affected by N, P, and K in the first year and by N and P during second year (Table 6). The increase in protein and total chlorophyll in response to N, P, and K were correlated with increases in photosynthesis. This relationship is consistent with chlorophyll serving as the main light-harvesting pigment for photosynthesis and with the observation that proteins associated with photosynthesis accumulate to extremely high levels, such as Rubisco and the light-harvesting chlorophyll *a/b*-binding proteins. Another reason may be that all of the three factors, especially N decreased stress induced damage by regulating enzymatic and non-enzymatic activities (Hussain et al., 2016). Thus, the plants were required to devote fewer resources to stress tolerance and therefore, they were able to invest more resources in processes, such as photosynthesis, that drive growth and development (Abid et al., 2016). N was reported to induce leaf development and photosynthetic efficiency (Dordas and Sioulas, 2008), P was reported to encourage carbon assimilation and the phosphorylation that enhances the regeneration of Rubisco (Liu et al., 2010), and K was reported to regulate stomata (Tsonev et al., 2011). We suggest that all of these factors contributed to higher protein and chlorophyll concentrations that we observed. Thus, our findings are consistent with previous work that documented increases in protein and chlorophyll levels following fertilization with N and P (Dordas and Sioulas, 2008; Nemadodzi et al., 2017).

## Conclusion

The results of this study demonstrated that different combinations of N, P, and K affected the morphology and physiology of overseeded perennial ryegrass on bermudagrass with a number of interactive effects. N, P, and K had positive effects on morphological traits with the exception of spring transition and physiological traits with the exception of WUE, and had interactive effects on ITP. The magnitudes of these nutrients effects on ITP followed the order of: N>P>K. Regarding the morphological attributes, growth parameters were mostly affected by fertilization with N and P, and qualitative parameters were affected by fertilization with N, P, and K. N and P delayed the spring transition while, K accelerated the spring transition. Fertilization with N, P, and K markedly increased *Pn, Tr, Gs*, levels of protein and total chlorophyll. Regression models between ITP and the three nutrients were established, and N, P, and K were optimized by simulation. The optimized scheme with maximum turf performance (ITP) was N: 30, P: 24, K: 9 g m^−2^ in first experiment and N: 30, P: 27, K: 6 g m^−2^ in the second experiment. Based on our results, we conclude that this approach can be effectively used to establish nutrient management strategies to maximize performance and yield. To cope with cold climates, turfgrasses management will depend not only on breeding new varieties that are well adopted to cold conditions, but also on the development of nutrient management practices, that will contribute to sustainable crop production.

## Author contributions

LC and SY: conceived the idea of the experiment; MI: performed the experiment and wrote manuscript; RL: revised and edited the manuscript; SF and TL: contributed to the collection of the data and statistical analysis; RL, SY, and LC: provided the technical guidance and editing support.

### Conflict of interest statement

The authors declare that the research was conducted in the absence of any commercial or financial relationships that could be construed as a potential conflict of interest.

## References

[B1] Abid M. Tian Z. Ata-Ul-Karim S. T. Cui Y. Liu Y. Zahoor R.. (2016). Nitrogen nutrition improves the potential of wheat (*Triticum aestivum* L.) to alleviate the effects of drought stress during vegetative growth periods. Front. Plant Sci. 7:981. 10.3389/fpls.2016.00981 27446197 PMC4927619

[B2] Ahmad I. Khan M. Qasim A. (2003). Growth and development of different turfgrasses as influenced by nitrogen application and leaf nitrogen contents. Int. J Agric. Biol. 5, 175–178.

[B3] Alderman P. Boote K. Sollenberger L. Coleman S. (2011). Carbohydrate and nitrogen reserves relative to regrowth dynamics of ‘Tifton 85’bermudagrass as affected by nitrogen fertilization. Crop Sci. 51, 1727–1738. 10.2135/cropsci2010.09.0516

[B4] Ashraf M. Ahmad A. McNeilly T. (2001). Growth and photosynthetic characteristics in pearl millet under water stress and different potassium supply. Photosynthetica 39, 389–394. 10.1023/A:1015182310754

[B5] Aulakh M. S. Malhi S. S. (2005). Interactions of nitrogen with other nutrients and water: effect on crop yield and quality, nutrient use efficiency, carbon sequestration, and environmental pollution. Adv. Agron. 86, 341–409. 10.1016/S0065-2113(05)86007-9

[B6] Baldi A. Lenzi A. Nannicini M. Pardini A. Tesi R. (2013). Growth and nutrient content of hybrid bermudagrass grown for nursery purposes at different nitrogen, phosphorus, and potassium rates. Horttechnology 23, 347–355.

[B7] Beard J. Menn W. (1988). The Texas System of Winter Overseeding. Grounds Maintenance (USA).

[B8] Cechin I. de Fátima Fumis T. (2004). Effect of nitrogen supply on growth and photosynthesis of sunflower plants grown in the greenhouse. Plant Sci. 166, 1379–1385. 10.1016/j.plantsci.2004.01.020

[B9] Çelebi Z. Andiç N. Yilmaz H. (2010). Determination of proper species mixtures for established turfgrass field in Van region. YYU. J. Agr. Sci. 20, 16–25.

[B10] Deng G. Liu L. J. Zhong X. Y. Lao C. Y. Wang H. Y. Wang B.. (2014). Comparative proteome analysis of the response of ramie under N, P and K deficiency. Planta 239, 1175–1186. 10.1007/s00425-014-2040-3 24573224

[B11] Din M. Zheng W. Rashid M. Wang S. Shi Z. (2017). Evaluating hyperspectral vegetation indices for leaf area index estimation of *Oryza sativa* L. at diverse phenological stages. Front. Plant Sci. 8:820. 10.3389/fpls.2017.00820 28588596 PMC5438995

[B12] Dordas C. A. Sioulas C. (2008). Safflower yield, chlorophyll content, photosynthesis, and water use efficiency response to nitrogen fertilization under rainfed conditions. Ind. Crop Prod. 27, 75–85. 10.1016/j.indcrop.2007.07.020

[B13] Duble R. L. (2001). Turfgrasses: Their Management and Use in the Southern Zone. College Station, TX: Texas A&M University Press.

[B14] Esmaeili S. Salehi H. Eshghi S. (2015). Silicon ameliorates the adverse effects of salinity on turfgrass growth and development. J. Plant Nutr. 38, 1885–1901. 10.1080/01904167.2015.1069332

[B15] Fahad S. Hussain S. Saud S. Hassan S. Tanveer M. Ihsan M. Z.. (2016). A combined application of biochar and phosphorus alleviates heat-induced adversities on physiological, agronomical and quality attributes of rice. Plant Physiol. Biochem. 103, 191–198. 10.1016/j.plaphy.2016.03.001 26995314

[B16] Fan J. Ren J. Zhu W. Amombo E. Fu J. Chen L. (2014). Antioxidant responses and gene expression in bermudagrass under cold stress. J. AM. Soc. Hortic. Sci. 139, 699–705. 10.1007/s10646-016-1696-9

[B17] Fredeen A. L. Raab T. K. Rao I. M. Terry N. (1990). Effects of phosphorus nutrition on photosynthesis in *Glycine max* (L.) Merr. Planta 181, 399–405. 10.1007/BF00195894 24196818

[B18] Giersch C. Robinson S. P. (1987). Regulation of photosynthetic carbon metabolism during phosphate limitation of photosynthesis in isolated spinach chloroplasts. Photosynth. Res. 14, 211–227. 10.1007/BF00032706 24430736

[B19] Grygierzec B. (2012). Productivity of selected grasses in mixtures with *Trifolium repens* L. at two levels of nitrogen fertilization. Fragm. Agron. 29, 31–36.

[B20] Guertal E. (2006). Phosphorus movement and uptake in bermudagrass putting greens. USGA Turfgrass Environ. Res. Online 5, 1–7.

[B21] Guertal E. Evans D. (2006). Nitrogen rate and mowing height effects on TifEagle bermudagrass establishment. Crop Sci. 46, 1772–1778. 10.2135/cropsci2006.01-0006

[B22] Guo X. Sun W. Wei W. Yang J. Kang Y. Wu J. (2009). Effects of NPK on physiological and biochemical characteristics of winter rapaseed in northwest cold and drought region. Acta Agric. Boreali-Occident. Sin. 2, 27.

[B23] Halder G. Dhawane S. Barai P. K. Das A. (2015). Optimizing chromium (VI) adsorption onto superheated steam activated granular carbon through response surface methodology and artificial neural network. Environ. Prog. Sustain. 34, 638–647. 10.1002/ep.12028

[B24] Hons F. McFarland M. L. Lemon R. G. Nichols R. L. Mazac F. Jr. Boman R. (2004). Managing Nitrogen Fertilizer in Cotton. College Station, TX: Texas A&M University Press.

[B25] Hussain S. Khan F. Cao W. Wu L. Geng M. (2016). Seed priming alters the production and detoxification of reactive oxygen intermediates in rice seedlings grown under sub-optimal temperature and nutrient supply. Front. Plant Sci. 7:439. 10.3389/fpls.2016.00439 27092157 PMC4820636

[B26] Jellicorse W. R. Richardson M. D. McCalla J. H. Karcher D. E. Patton A. J. Boyd J. W. (2012). Seeded bermudagrass establishment in an overseeded perennial ryegrass stand as affected by transition herbicide and seeding date. Appl. Turfgrass Sci. 9 10.1094/ATS-2012-0721-01-RS

[B27] Jiang Y. Huang B. (2000). Effects of drought or heat stress alone and in combination on Kentucky bluegrass. Crop Sci. 40, 1358–1362. 10.2135/cropsci2000.4051358x

[B28] Larcher W. (2003). Plants under stress, in Physiological Plant Ecology, Ecophysiology and Stress of Functional Groups, 4th Edn, (Berlin; Heidelberg: Springer), 345–450.

[B29] Liu H. Hu C. Sun X. Tan Q. Nie Z. Hu X. (2010). Interactive effects of molybdenum and phosphorus fertilizers on photosynthetic characteristics of seedlings and grain yield of Brassica napus. Plant Soil 326, 345–353. 10.1007/s11104-009-0014-1

[B30] Mazur A. Wagner D. (1987). Influence of aeration, topdressing, and vertical mowing on overseeded bermudagrass putting green turf. HortScience 22, 1276–1278.

[B31] McCarty L. B. Miller G. (2002). Managing Bermudagrass Turf: Selection, Construction, Cultural Practices, and Pest Management Strategies. Ann Arbor, MI: John Wiley & Sons.

[B32] Montgomery D. C. (2001). Design and Analysis of Experiments. New York, NY: John Wiley & Sons.

[B33] Munshaw G. Ervin E. Parrish D. Shang C. Askew S. Zhang X. (2007). Influence of late-season iron, nitrogen, and seaweed extract on fall color retention and cold tolerance of four bermudagrass cultivars. Crop Sci. 47, 463–463. 10.2135/cropsci2007.01.0001er

[B34] Nemadodzi L. Araya H. Nkomo M. Ngezimana W. Mudau N. F. (2017). Nitrogen, phosphorus and potassium effects on the physiology and response biomass yield of baby spinach *(Spinacia oleracea* L.). J. Plant Nutr. 40, 2033–2044. 10.1080/01904167.2017.1346121

[B35] Pompeiano A. Guglielminetti L. Volterrani M. (2011). Freeze tolerance of *Zoysia matrella* (L.) Merrill as affected by late-season nitrogen application, and changes in carbohydrates during cold acclimation. Plant Biosyst. 145, 885–892. 10.1080/11263504.2011.623191

[B36] Rajasekar M. Nandhini D. U. Swaminathan V. Balakrishnan K. (2017). A review on role of macro nutrients on production and quality of vegetables. Int. J. Chem. Sci. 5, 304–309.

[B37] Razmjoo K. Kaneko S. (1993). Effect of fertility ratios on growth and turf quality of perennial ryegrass (*Lolium prenne* L.) in winter. J. Plant Nutr. 16, 1531–1538. 10.1080/01904169309364630

[B38] Richardson M. (2004). Morphology, turf quality, and heat tolerance of intermediate ryegrass. HortScience 39, 170–173.

[B39] Rodriguez I. R. Miller G. L. McCarty L. (2002). Bermudagrass establishment on high sand-content soils using various NPK ratios. HortScience 37, 208–209.

[B40] Römheld V. Kirkby E. A. (2010). Research on potassium in agriculture: needs and prospects. Plant Soil 335, 155–180. 10.1007/s11104-010-0520-1

[B41] Schlossberg M. J. Schmidt J. P. (2007). Influence of nitrogen rate and form on quality of putting greens cohabited by creeping bentgrass and annual bluegrass. Agron. J. 99, 99–106. 10.2134/agronj2006.0136

[B42] Shi H. Jiang C. Ye T. Tan D.-X. Reiter R. J. Zhang H.. (2014a). Comparative physiological, metabolomic, and transcriptomic analyses reveal mechanisms of improved abiotic stress resistance in bermudagrass [*Cynodon dactylon* (L). Pers.] by exogenous melatonin. J. Exp. Bot. 66, 681–694. 10.1093/jxb/eru373 25225478 PMC4321537

[B43] Shi H. Ye T. Zhong B. Liu X. Chan Z. (2014b). Comparative proteomic and metabolomic analyses reveal mechanisms of improved cold stress tolerance in bermudagrass (*Cynodon dactylon* (L.) Pers.) by exogenous calcium. J. Integr. Plant Biol. 56, 1064–1079. 10.1111/jipb.12167 24428341

[B44] Snyder G. H. Cisar J. L. (2000). Nitrogen/potassium fertilization ratios for bermudagrass turf. Crop Sci. 40, 1719–1723. 10.2135/cropsci2000.4061719x

[B45] Stanford R. White R. Krausz J. Thomas J. Colbaugh P. Abernathy S. (2005). Temperature, nitrogen and light effects on hybrid bermudagrass growth and development. Crop Sci. 45, 2491–2496. 10.2135/cropsci2005.0070

[B46] Tsonev T. Velikova V. Yildiz-Aktas L. Gürel A. Edreva A. (2011). Effect of water deficit and potassium fertilization on photosynthetic activity in cotton plants. Plant Biosyst. 145, 841–847. 10.1080/11263504.2011.560199

[B47] Turgeon A. J. (2008). Turfgrass Management, 8th Edn. Upper Saddle River, NJ; Columbus, OH: Pearson Prentice Hall.

[B48] Tyler N. Gusta L. Fowler D. (1981). The influence of nitrogen, phosphorus and potassium on the cold acclimation of winter wheat (*Triticum aestivum* L.). Can. J. Plant Sci. 61, 879–885. 10.4141/cjps81-131

[B49] Uleberg E. Hanssen-Bauer I. van Oort B. Dalmannsdottir S. (2014). Impact of climate change on agriculture in Northern Norway and potential strategies for adaptation. Clim. Change 122, 27–39. 10.1007/s10584-013-0983-1

[B50] Vance C. P. (2001). Symbiotic nitrogen fixation and phosphorus acquisition. Plant nutrition in a world of declining renewable resources. Plant Physiol. 127, 390–397. 10.1104/pp.010331 11598215 PMC1540145

[B51] Verdenal A. Combes D. Escobar-Gutierrez A. J. (2008). A study of ryegrass architecture as a self-regulated system, using functional-structural plant modelling. Funct. Plant Biol. 35, 911–924. 10.1071/FP08050 32688842

[B52] Wang J. Hui D. Ren H. Liu Z. Yang L. (2013). Effects of understory vegetation and litter on plant Nitrogen (N), Phosphorus (P), N: P Ratio and their relationships with growth rate of indigenous seedlings in subtropical plantations. PLoS ONE 8:84130. 10.1371/journal.pone.0084130 24386340 PMC3873957

[B53] Wang S. Yang J. F. Han X. R. Liu X. H. Zhan X. M. Liu S. G. (2008). Effect of fertilizer application on photosynthetic traits of spring maize. Soil Fert. Sci. Chin. 6, 23–27.

[B54] Webster D. Ebdon J. (2005). Effects of nitrogen and potassium fertilization on perennial ryegrass cold tolerance during deacclimation in late winter and early spring. HortScience 40, 842–849.

[B55] Wise R. Olson A. Schrader S. Sharkey T. (2004). Electron transport is the functional limitation of photosynthesis in field-grown pima cotton plants at high temperature. Plant Cell Environ. 27, 717–724. 10.1111/j.1365-3040.2004.01171.x

[B56] Yang Z. Miao Y. Yu J. Liu J. Huang B. (2014). Differential growth and physiological responses to heat stress between two annual and two perennial cool-season turfgrasses. Sci. Hortic. Amsterdam 170, 75–81. 10.1016/j.scienta.2014.02.005

[B57] Zlatev Z. Lidon F. C. (2012). An overview on drought induced changes in plant growth, water relations and photosynthesis. Emir. J. Food Agric. 24, 57–72. 10.9755/ejfa.v24i1.10599

